# HLA Class I Allele Frequencies in Southern Iranian Women with Breast Cancer 

**Published:** 2013-02

**Authors:** Mahboobeh Razmkhah, Abbas Ghaderi

**Affiliations:** 1 Shiraz Institute for Cancer research, Medical School, Shiraz University of Medical Sciences, Shiraz, Iran; 2 Department of Immunology, Medical School, Shiraz University of Medical Sciences, Shiraz, Iran

**Keywords:** Breast cancer, HLA alleles, Malignancy

## Abstract

***Objective(s):*** Breast cancer is the leading cause of cancer-related death in women worldwide. It has been revealed that elevated risk for malignancy may be associated with certain HLA alleles. This study was performed to assess the association of HLA class I alleles with breast cancer in women in Southern Iran.

***Materials and Method: ***Eighty nine patients included for analyzing the HLA class I alleles frequency using complement dependent cytotoxicity microassay and results were compared to 86 gender-matched healthy volunteers.

***Results:*** There were significantly more patients with A24(9) allele than those of healthy individuals (38.2% versus 16.3%) (P-value=0.002). In contrast, HLA-A1 had significantly much less expression in the patient group compared to the controls (P- value=0.04).

***Conclusion:*** A24(9) allele appears to be one of the factors increasing an individual’s the susceptibility to breast cancer in our population but further investigation might be required.

## Introduction

It has frequently been reported that certain human leukocyte antigen (HLA) class I and II alleles may be associated with an increased risk of various type of malignancies (1,2). The positive association of HLA-A24 (9) with higher stages of lung cancer (3),-DRB1*12 (4), -DRB3*02 (5), -B7 (6,7), and -A24-CW7 (8) with breast cancer and HLA-DQB1* 0602 with gastric cancer (9) have been reported previously. Ghaderi *et al* have reported a positive relationship between HLA-DRB1*12 and breast cancer in Southern Iranian patients. In contrast, DQ13* 03032 and –DRB1*11 alleles may represent resistance to breast cancer (4). The expression of specific HLA class I alleles and positive associations of HLA class II have also been reported in human tumors of the pancreas (10).

Allele-specific down-regulation of HLA class I was formerly revealed in different cancer cells (11, 12). Invasive tumors lose certain HLA antigens at a frequency of 40-90%. Interestingly, the altered expression of HLA class I molecules in tumors was associated with tumor metastasis (13). 

HLA class I molecules are considered as regulators of CD8(+) T-cell and natural killer cells (NK). It has been illustrated that all HLA class I loci and alleles vary extensively for their interaction with killer-cell immunoglobulin-like receptors (KIRs) on NK cells (14). Thus, altered HLA phenotypes are apparently associated with a modified immune response against tumor cells (13). 

Assessing the nature of HLA alleles is an important endeavor since there is evidence to support peptide vaccination strategies for different type of cancers (15-23). Thus, the present study was performed to determine the probable association of HLA-A and -B alleles in Southern Iranian women with breast cancer compared to healthy individuals. Results may have important implications for better understanding of anti-tumor immune responses.

## Material and Methods

*Subjects*

Eighty nine patients diagnosed with breast carcinoma and 86 healthy women participated in this study following informed consent. Healthy volunteers had no history of autoimmune and malignancy disorders. The age of the patients ranged from 23 to 80 years, whereas healthy controls were aged 30-58 years.

Breast cancer was evidenced from surgical and pathological reports. Briefly, 90% of patients were diagnosed with infiltrative ductal carcinoma, 8.9% with modularly carcinoma and 0.6% with in situ and papillary carcinoma. Almost 53.2% and 60.1% of patients expressed estrogen and progesterone receptors (ER and PR), respectively. 58.3% of patients had lymph node involvement and 78.5% had preneural, vascular or lymphatic invasion.

*HLA typing using Complement Dependent Cytotoxicity (CDC) Assay*

*Preparing the anti-HLA class I antisera coated plates*

Five µl mineral oil was initially added to each well of 96 well plates in order to prevent the evaporation of antiserum which was added later. Subsequently, 1 µl of commer cially prepared antisera (Inno-Tron, Germany) against different HLA-I alleles was added to each well (each well should contain a particular antiserum). Plates were maintained at -20˚C.

Sampling and separation of peripheral blood mononuclear cells (PBMCs)

Peripheral Blood Mononuclear Cells (PBMCs) were prepared from 3-5 ml heparinized peripheral blood by density gradient centrifugation over Ficoll-hypaque (Biosera, UK). Cells were washed three times with Hank’s solution, then the supernatant was poured out and 1 ml of Hank’s solution was added into the tube and mixed gently. Separated lymphocytes were isolated by a homocytometer chamber and adjusted to 3000-5000 cells/µl.

CDC procedure

Prior to test initiation, the plates were put in room temperature (RT) which resulted in melting of the antisera. Then 1µl of PBMCs was added to each well with a Hamilton syringe and put in RT for 1 hr duration. Subsequently, 5µl of complement (pooled rabbit serum) was added to each well and again put in RT for 1 hr to allow cytotoxicity to occur. In the last step, 2 µl of 3.5% eosin (Sigma, USA) was poured in each well and by regarding the controls; results were rapidly analyzed by inverted microscope on the basis of the percentage of dead cells. Those wells with more than 50% dead cells were considered positive for that specific antiserum.

**Table 1:** The percentage of different HLA alleles in women diagnosed with breast cancer and healthy volunteers

HLA	Patients (%)	Controls (%)	*P*- value	HLA	Patients	Controls (%)	*P*- value
A1	11.2	24.4	0.04*	B16	5.6%	10.5	0.4
A2	37.1	35	0.8	B35	37%	35	0.9
A23(9)	0	3.5	0.2	B57 (17)	3.4%	2.3	0.9
A24(9)	38.2	16.3	0.002*	B22	3.4%	11.6	0.07
A10	9	14	0.4	B21	5.6%	8.1	0.7
A11	14.6	23.3	0.2	B17	4.5%	4.7	0.8
A28	11.2	21	0.1	B51(5)	31.5%	33.7	0.9
A29	2.3	5.8	0.4	B5	9%	7	0.8
A32	15.7	11.6	0.5	B27	3.4%	1.2	0.6
A33	6.7	1.2	0.1	B37	1.1%	2.3	0.9
B7	7.9	9.3	0.9	B40	10.11%	3.5	0.2
B8	6.7	15.1	0.1	B44 (12)	9%	16.3	0.2
B13	3.4	9.3	0.2	B42	1.1%	2.3	0.9
B14	4.5	9.3	0.3	B41	4.5%	3.5	0.9

Statistical analysis

Data was analyzed by χ2-test using EPI-Info 2002 and SPSS version 11.5. Results were considered significant at *P-*value less than 0.05. 

## Results

The frequency of HLA class I alleles

As presented in Table 1, HLA-A24(9) allele had higher expression in patients than in controls. It was detected in 34 (38.2%) patients while 14 (16.3%) healthy women expressed this allele. This difference was statistically significant (*P *value=0.002). In contrast, the frequency of HLA-A1 had statistically significant lower expression in patients than controls, 11.2% versus 24.4%, *P* value=0.04. There were no statistically significant difference in the frequency of other HLA-A and B alleles between patients and controls.

Association of HLA alleles and the clinicopathological status of patients

There were no association between different HLA alleles and the important prognostic factors in breast cancer such as ER and PR expressions and pathological stages. 

## Discussion

Specific HLA alleles undoubtedly play essential roles in cancer predisposition, through the modulation of innate and adaptive immune responses (24). In the present study, the frequency of HLA class A and B alleles were investigated in Southern Iranian women with breast cancer. The results revealed that there were significantly higher number of patients with A24(9) allele than those of healthy individuals. In contrast, HLA-A1 had significantly much less expression in the patient group compared to the controls. Consistent with our finding on A24(9) allele as a predisposing factor for breast cancer, the same association have been reported in glioma (25), testicular cancer (26), breast cancer in a Japanese individuals (8), and thymoma in a German population of myastenia gravis (27). In contrast, Wei *et al* have suggested HLA-A*24 as a protective gene marker for chronic myelogenous leukemia (CML) in a Chinese population (28).

There is extensive evidence that human tumor cells reduced or lost HLA class I antigen expression at a very high frequency (13). Altered HLA class-I represent a major mechanism that helps tumor cells to escape T cell mediated immune response (10,29). HLA allelic loss is observed in approximately 15% - 51% of all tumor types (13). Gharesi *et al* have reported the allelic loss of HLA class I in cryopreserved tissue sections of the breast in Southern Iranian individuals with breast carcinoma. They noticed that among different HLA class I alleles A24(9) , A11, A28 of locus A and B53, B18, B13, and B14 of locus B are the highest frequent alleles with allelic loss (unpublished data). With respect to the results of the present study, the allelic loss of A24(9) in breast tissue of breast cancer patients may be the main reason for the susceptibility of A24(9) carriers to breast cancer in our population.

It has been reported that HLA-A24(9) is secreted further than other HLA-I molecules (30,31) especially in women during the first half of the menstrual cycle (32). In this context, it is important to note that soluble HLA (sHLA)-I antigens may have a tolerogenic consequence and may be a mechanism of tumor escape (33), through down regulating NK cell activation and inducing soluble FasL secretion (34). Based on these reports, we hypothesized that increasing sHLA-I in individuals with A24(9) allele may influence the susceptibility of carriers to different types of malignancies by downregulating immune responses and contributing to tumor escape.

 Considering the frequency of A24 allele in our breast cancer subjects, HLA-A24 specific peptides may provide important implications in peptide vaccination strategy for immunotherapy of these patients in our area. Furthermore, HLA-A24 specific peptides of HER2/neu (15), tyrosinase in melanoma (16), CEA and MAGE-3 in gastrointestinal malignancies (17,18), SART-1 in oral squamous cell carcinoma (19), MUC5AC in pancreatic cancer (35), Eras in gastric cancer (36) and P53 in bladder cancer (20,21) had previously been used.

It has long been recognized that cancer is a multigenic disease. Thus, the frequency of an HLA allele or haplotype in a population and its linkage to other genes, in particular tumor suppressor genes,or genes of the components of immune system, should be considered when investigating the etiology of breast cancer. Accordingly, it has been reported that A24(9) allele may have linkage disequilibrium with CW7 in breast cancer patients (8) and also with C4A, C4B and BF in Korean and Australian populations (29,37,38). 

## Conclusion

Overall, the results of this study determined that the HLA-A24(9) allele is a gene marker for the susceptibility to breast cancer in Southern Iranian population. Further investigation particularly with high resolution typing procedures on a larger population and the evaluation of HLA haplotypes is required for better elucidation. 

## References

[1] Hildesheim A, Wang SS (2002). Host and viral genetics and risk of cervical cancer: a review. Virus Res.

[2] Hassen E, Nahla G, Bouaouina N, Chouchane L (2010). The human leukocyte antigen class I genes in nasopharyngeal carcinoma risk. Mol Biol Rep.

[3] Nagata Y, Hanagiri T, Mizukami M, Kuroda K, Shigematsu Y, Baba T (2009). Clinical significance of HLA class I alleles on postoperative prognosis of lung cancer patients in Japan. Lung Cancer.

[4] Ghaderi A, Talei A, Gharesi-Fard B, Farjadian Sh, Amirzargar A, Vasei M (2001). HLA-DRB1 allele and the susceptibility of Iranian patients with breast cancer. Pathol Oncol Res.

[5] Chaudhuri S, Cariappa A, Tang M, Bell D, Haber DH, isselbacher KJ (2000). Genetic susceptibility to breast cancer: HLA DQ*03032 and HLA DRB1*11 may represent protective alleles. Proc Natl Acad Sci USA.

[6] Gourley C, Thornton C, Massie C, Prescott RJ, Turner M, Leonard RC (2003). Is there a relationship between HLA type and prognostic factors in breast cancer. Anticancer Res.

[7] Lavado R, Benavides M, Villar E, Ales I, Alonso A, Caballero A (2005). The HLA-B7 allele confers susceptibility to breast cancer in Spanish women. Immunol Lett.

[8] Yokoe T, Ishida T, Ogawa T, Iino Y, Izuo M (1990). Relationship of breast cancer and HLA in Japanese females. Gan No Rinsho.

[9] Wu MS, Hsieh RP, Huang SP, Chang YT, Lin MT, Chang MC (2002). Association of HLA-DQB1(*)0301 and HLA-DQB1(*)0602 with different subtypes of gastric cancer in Taiwan. Jpn J Cancer Res.

[10] Ryschin E, Autschbach F, Eisold S, Klar E, Buchler MW, Schmidt J (2003). Expression of HLA class I/II antigens and T cell immune response in human neuroendocrine tumors of the pancreas. Tissue Antigens.

[11] Hiraki A, Fujii N, Murakami T, Kiura K, Aoe K, Yamane H (2004). High frequency of allele-specific down-regulation of HLA class I expression in lung cancer cell lines. Anticancer Res.

[12] Belov K (2011). The role of the Major Histocompatibility Complex in the spread of contagious cancers. Mamm Genome.

[13] Algarra I, Cabrera T, Garrido F (2000). The HLA crossroad in tumor immunology. Human Immunol.

[14] Kulkarni S, Martin MP, Carrington M (2008). The Yin and Yang of HLA and KIR in human disease. Semin Immunol.

[15] Ikuta Y, Okugawa T, Furugen R, Nagata Y, Takahashi Y, Wang L (2000). A HER2/NEU-derived peptide, a K(d)-restricted murine tumor rejection antigen, induces HER2-specific HLA-A2402-restricted CD8(+) cytotoxic T lymphocytes. Int J Cancer.

[16] Bettinotti MP, Norris RD, Hackett JA, Thompson CO, Simonis TB, Stroncek D (2000). Frequency of human leukocyte antigen-A24 alleles in patients with melanoma determined by human leukocyte antigen-A sequence-based typing. J Immunother.

[17] Matsuda K, Tsunoda T, UmanoY, Tanimuta H, Nukaya L, Takesako K (2004). Enhancement of cytotoxic T-lymphocyte responses in patients with gastrointestinal malignancies following vaccination with CEA peptide-pulsed dendritic cells. Cancer Immunol Immunother.

[18] Hao X, Shao Y, Ren X, Liu H, Xu Q, Li H (2002). Induction of specific CTL by MAGE-3/CEA peptide-pulsed dendritic cells from HLA-A2/A24(+) gastrointestinal cancer patients. J Cancer Res Clin Oncol.

[19] Kumamaru W, Nakamura S, Kadena T, Yamada A, Kawamura E, Sasaki M (2004). T-cell receptor V beta gene usage by T cells reactive with the tumor-rejection antigen SART-1 in oral squamous cell carcinoma. Int J Cancer.

[20] Eura M, Chikamatsu K, Katsura F, Obata A, SabaoY, Takiguchi M (2000). A wild-type sequence P53 peptide presented by HLA-A24 induces cytotoxic T lymphocytes that recognize squamous cell carcinoma of the head and neck. Clin Cancer Res.

[21] Ferries E, Connan F, Pages F, Gaston J, Hagnere AM, Vieillefond A (2001). Identification of p53 peptides recognized by CD8 (+) T lymphocytes from patients with bladder cancer. Hum Immunol.

[22] Gritzapis AD, Voutsas IF, Lekka E, Tsavaris N, Missitzis I, Sotiropoulou P (2008). Identification of a novel immunogenic HLA-A*0201-binding epitope of HER-2/neu with potent antitumor properties. J Immunol.

[23] Morse MA, Secord AA, Blackwell K, Hobeika AC, Sinnathamby G, Osada T (2011). MHC class I-presented tumor antigens identified in ovarian cancer by immunoproteomic analysis are targets for T-cell responses against breast and ovarian cancer. Clin Cancer Res.

[24] Zhao M, Cai H, Li X, Zheng H, Yang X, Fang W (2012). Further evidence for the existence of major susceptibility of nasopharyngeal carcinoma in the region near HLA-A locus in Southern Chinese. J Transl Med.

[25] Nitta T, Ebato M, Sato K (1994). Association of malignant glioma with the human leukocyte antigen, HLA-A24(9). Neurosurg Rev.

[26] Ishikawa K, Nakano H, Tsukamoto, Fujioka T, Nagakubo I (1996). Testicular cancer in father and son. Int J Urol.

[27] Machens A, Löliger C, Pichlmeier U, Emskötter Th, Busch C, Izbicki JR (1999). Correlation of thymic pathology with HLA in Myasthenia Gravis. Clin Immunol.

[28] Wei L, Xiao LL, Wu XY, Lin Q, Dong M, Wen JY (2008). Expression and analysis of HLA-A, B and DRB1 genes in patients with chronic myelogenous leukemia in Guangdong area. Zhongguo Shi Yan Xue Ye Xue Za Zhi.

[29] Hiraki A, Ikeda K, Yoshino T, Kaneshige T, Kiura K, Kunisada T (2001). Tumor-specific cytotoxic T lymphocyte responses against chondrosarcoma with HLA haplotype loss restricted by the remaining HLA class I allele. Biochem Biophys Res Commun.

[30] Adamashvili IM, Fraser PA, McDonald JC (1996). Association of serum concentration of soluble class I HLA with HLA allotypes. Trasplantation.

[31] Shimura T, Hagihara M, Yamamoto K, Takebe K, Munkhbat B, Ogoshi K (1994). Quantification of serum-soluble HLA class I antigens patients with gastric cancer. Hum Immunol.

[32] Zavazava N, Wobst B, Ferstl R, Muller-Ruchholtz W (1994). Soluble MHC class I molecules in human body fluids. J Clin Lab Anal.

[33] Spaggriari MG, Contini P, Dondero A, Carosio R, Puppo F, Indiveri F (2002). Soluble HLA class I induces NK cell apoptosis upon the engagement of killer-activating HLA class I receptor through FASL-FAS interaction. Blood.

[34] Puppo F, Contini P, Ghio M, Brenci S, Scudeletti M, Filaci G (2000). Soluble human MHC class I molecules induce soluble Fas ligand secretion and trigger apoptosis in activated CD8+ Fas (CD95)+ T lymphocytes. Int Immunol.

[35] Yamazoe S, Tanaka H, Iwauchi T, Yoshii M, Ito G, Amano R (2011). Identification of HLA-A*0201- and A*2402-restricted epitopes of mucin 5AC expressed in advanced pancreatic cancer. Pancreas.

[36] Iwauchi T, Tanaka H, Yamazoe S, Yashiro M, Yoshii M, Kubo N (2011). Identification of HLA-A*2402-restricted epitope peptide derived from ERas oncogene expressed in human scirrhous gastric cancer. Cancer Sci.

[37] Park KS, Park MH, Juji T, Tokunaga K (1996). Complement C4A, C4B and BF haplotype in Koreans. Tissue Antigens.

[38] Tokunaga K, Zhang WJ, Christiansen FT, Dawkins RL (1991). The genomic structure of two ancestral haplotype carrying C4A duplications. Immunogenetics.

